# Myositis ossificans in a child athlete: a case study

**DOI:** 10.17159/2078-516X/2022/v34i1a14931

**Published:** 2022-01-01

**Authors:** R Sapire, R Nenova, P Gounder, A Rampersad, V Maboho, N Nhlapo, K Tibatshi, S Rampurtab, AI Ranchod, RT Saggers, J Patricios

**Affiliations:** 1 Unit for Undergraduate Medical Education, Faculty of Health Sciences, University of the Witwatersrand, Johannesburg, South Africa; 2 Wits Sport and Health (WiSH), School of Clinical Medicine, Faculty of Health Sciences, University of the Witwatersrand, Johannesburg, South Africa; 3 Department of Diagnostic Radiology, University of the Witwatersrand, Johannesburg, South Africa; 4 Department of Paediatrics and Child Health, Charlotte Maxeke Johannesburg Academic Hospital and School of Clinical Medicine, Faculty of Health Sciences, University of the Witwatersrand, Johannesburg, South Africa; 5 Netcare Waterfall Sports Orthopaedic Surgery, Johannesburg, South Africa

**Keywords:** traumatic, benign, malignant, extracorporeal shock-wave therapy, osteosarcoma

## Abstract

**Background:**

A 13-year-old female athlete presented with a painful lesion in her right buttock for which she had been receiving physiotherapy. It was keeping her from participating in sports.

**Aim:**

To report on a case of traumatic myositis ossificans in a child athlete – including the presentation, investigations, management, and outcome.

**Findings:**

Palpation of the right buttock indicated a tender mass. Investigation by musculoskeletal ultrasound detected a large hypoechoic lesion. An MRI revealed patterns of calcification that were inconclusive in differentiating between a malignant or benign lesion. Macroscopic and microscopic histological examination, as well as immunohistochemistry, were consistent with myositis ossificans (MO), a non-malignant condition. The patient improved remarkably within three months of treatment with rest, non-steroidal anti-inflammatory drugs (NSAIDs) and extracorporeal shock wave therapy (ESWT).

**Implications:**

Accurate differentiation of myositis ossificans from other benign and malignant soft tissue lesions may require histological evaluation in addition to a comprehensive radiological workup. Successful treatment with the patient being able to return to a pain-free and active state is achievable. Extracorporeal shock-wave therapy can play an important role in the management of this condition and should be considered when presented with a case of MO.

## Case report

Myositis ossificans (MO) is a rare pathological disorder characterised by the accretion of non-neoplastic bone in skeletal muscle and surrounding soft tissue.^[1]^ Three types have been classified: hereditary MO (MO progressiva, a rare autosomal dominant condition), non-traumatic MO (associated with burns, haemophilia and neurological conditions) and traumatic MO (MO circumscripta, associated with direct or repetitive trauma). Traumatic MO is the most common – occurring in approximately 60–75% of cases – and is often secondary to sports-related impacts.^[2]^ Following traumatic incidents, the lesion arises from an inflammatory reaction that causes endothelial to mesenchyme transition (EndMT) in vascular endothelial cells. The cells differentiate into chondrocytes, which then undergo endochondral bone formation or osteoblasts directly resulting in localised ossification.^[1]^

MO usually presents as a warm, tender swelling with overlying erythema and progresses to a palpable osseous mass with maturation. Symptoms include joint and muscle stiffness, pain on movement and a decreased range of motion. Commonly affected sites are the arm flexor and thigh extensor muscles.^[1]^ MO is rare in young children, occurring mostly in adolescents and young adults. On examination and radiological assessment, early MO presents with similar patterns to that of an osteosarcoma.^[3]^

Treatment modalities range from conservative therapies, such as rest, ice and elevation, with concurrent use of non-steroidal anti-inflammatories, radiotherapy, extracorporeal shock-wave therapy and acetic acid phonophoresis to more aggressive surgical approaches, which are usually only considered after 6 – 12 months of unsuccessful conservative therapy.^[3]^

### History

A 13-year-old female developed a large painful lesion in her right buttock. Pain radiated from the lesion to the posterior thigh and worsened at night, with no associated constitutional symptoms. The patient had been unable to participate in her usual sporting activities (namely, horse riding, hockey and tennis) and was receiving physiotherapy for the pain which was unresponsive to therapy. She had no recollection of trauma directly related to the site of the lesion.

### Physical examination

On general examination, the patient was healthy but in some discomfort upon sitting. On localised examination, there was a tender, palpable, golf ball-like mass in her right gluteal muscle with overlying erythema.

### Investigations

**Fig. 1:**
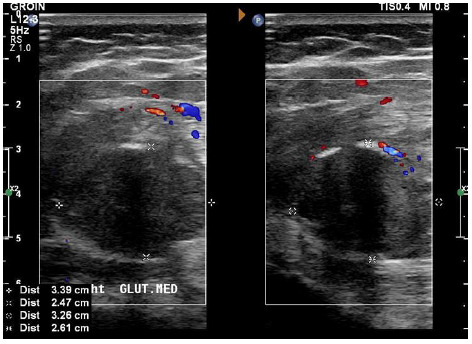
Doppler Ultrasound - Mass (70 x 45 x 35mm) identified within the right gluteus musculature. Mildly increased vascularity seen within the mass and at the periphery. Possible calcifications seen within the lesion.

Ultrasonography demonstrated a hypoechoic heterogeneous mass (70 x 45 x 35mm) in the proximal third of the right gluteus maximus muscle belly. Within this mass, there were areas that resembled the typical appearance for proliferative myositis. However, there was a solid more hypoechoic mass (35 x 25 x 33mm) which demonstrated two calcifications within the deep part of the larger mass identified. The muscle architecture, superficial and deep, appeared normal. A colour Doppler ultrasound showed mildly increased vascularity within the mass and peripherally (see Figure 1). Magnetic resonance imaging (MRI) was indicated, as a malignant soft tissue lesion could not be excluded by ultrasound.

**Fig. 2:**
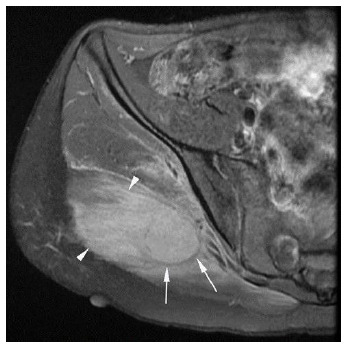
Axial MRI pelvis – significant surrounding oedema (arrowheads), extending laterally to the greater trochanter and medially into the sciatic notch. A rim of hypointense calcifications (arrows) seen at the periphery of the lesion. Axial measurement is 3.4 x 2.5cm.

A multiparametric pre- and post-contrast 1.5T MRI scan of the pelvis was obtained five days after presentation. There was a heterogeneously enhancing mass with thin peripheral calcification within the right gluteus maximus, with significant surrounding oedema (see Figure 2). These findings favoured the intermediate phase of MO as the primary differential diagnosis but could not definitively exclude malignancies, along with lymphoma, osteosarcoma and rhabdomyosarcoma.^[4]^ X-ray findings of the pelvis were suggestive of early rim ossification that occurs in a progressive MO lesion.

The blood results from the day of presentation showed normal red cell indices, normal absolute leukocyte values and moderate thrombocytosis. C-reactive protein, creatinine kinase and renal function were normal.

### Histology

Due to the uncertainty of the diagnosis and potential risk of a malignant lesion, the patient was referred to an orthopaedic surgeon with a specific interest in soft tissue lesions. A biopsy was taken and sent for histological evaluation. Macroscopic examination showed three gritty soft tissue fragments, with the largest fragment measuring 15 x 5 x 3mm. Immunohistochemistry on a population of spindle cells was performed. The immunoprofile, consisting of a moderate proliferation index and a positive smooth muscle actin marker, was compatible with the myofibroblastic nature of the proliferating cells and consistent with MO.

Microscopic examination of the paraffin sections showed subcutaneous fibroadipose connective tissue, skeletal muscle and an area of new bone formation in conjunction with a cellular spindle cell proliferation. The spindle cells exhibited a fibroblastic appearance. The peripheral bony trabeculae appeared relatively mature with clear osteoblastic rimming and orderly maturation. There was an impression of zonation within the lesion. Occasional normal mitotic figures were seen in the specimen. These findings were consistent with the reactive process seen in MO. There was no histological evidence of malignancy. Diagnosis of MO was then confirmed.

### Management

Following the diagnosis of MO, further interrogation of the patient’s history led to her revealing that she rode horses and had fallen three months before presentation, possibly on to her buttock.

She was advised to stop physiotherapy and all sporting activities to prevent exacerbation. The lesion was monitored for changes. A Central Sensitivity Inventory (CSI) self-report was used to quantify pain hypersensitivity. Oral indomethacin of 75mg daily was administered to reduce pain, stiffness and inflammation. A ring cushion was recommended to alleviate pressure to the area when sitting. Once tenderness had improved, extracorporeal shock-wave therapy (ESWT), under local anaesthesia, was performed on three occasions. Discharge and return to all activities and sports was granted three weeks after initial ESWT.

### Follow-up imaging

**Fig. 3:**
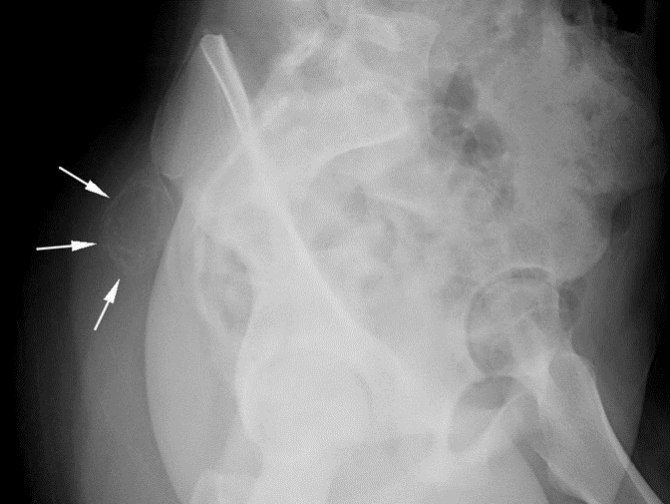
X-ray pelvis **–** typical peripheral ossification of the mature lesion (arrows).

After initiation of treatment the patient was followed up clinically and with imaging. After two months, X-rays remained unchanged, indicating stability of the lesion. Four months later, oblique X-ray imaging confirmed ossification and a slight reduction in size of the lesion (see Figure 3).

**Fig. 4:**
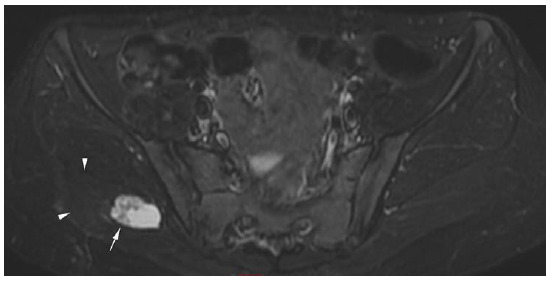
Axial MRI pelvis – complete resolution of soft tissue oedema (arrowheads) surrounding the mature lesion (arrow). Central soft tissue T2 hyperintense and T1 isointense. Axial measurement is 3.1 x 2cm.

**Fig. 5:**
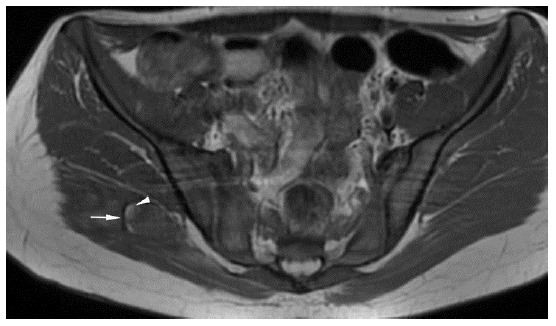
Axial MRI pelvis – fat signal present at periphery of the lesion (arrowhead) adjacent to the rim calcification (arrow) consistent with ossification.

A follow-up MRI six months after diagnosis showed resolution of the surrounding oedema. The central lesion remained but with an increased rim of calcification and fat-based tissue (likely representing marrow fat related to developing ossification) immediately adjacent to the calcification (see Figure 4 and Figure 5). The lesion was slightly smaller in size compared to previous measurements (see Figure 2). Furthermore, there was no fluid in the sacroiliac joints, and no other abnormalities within the hip joint and bursae. These MRI findings corresponded with the evolution of the lesion from the subacute or intermediate phase to the chronic phase.

### Outcome

Two months after the initiation of treatment, the patient’s discomfort with walking and night pains had lessened. She was able to resume low-intensity exercise, hop on the right leg, and do lunges without pain. The lesion was still palpable but reduced in size and less tender. After three months of treatment, she could tolerate moderate- to high-intensity exercise with no pain. The patient showed a complete recovery and was able to return to her usual sporting activities. At the time of writing, there were no signs of recurrence.

## Discussion

MO typically presents as an “inflammatory, rapidly growing and painful muscular mass”.^[4]^ The patient being reported on presented typically with a tender, palpable, osseous mass with overlying erythema.^[1]^

Of the three types of MO identified previously (traumatic, non-traumatic, hereditary), the traumatic pattern best fits in this patient. Athletes may sustain injury by either trauma (projectile or contact) or overuse, both of which have been linked to MO.^[1]^ The patient fell off a horse three months prior to initial presentation resulting in blunt force trauma to the affected area.

This case was selected to demonstrate that MO is a rare condition and can present similarly to malignant lesions, creating a diagnostic dilemma and emphasising the importance of excluding this differential. It also highlights the importance of conducting a thorough workup, from history and examination to a directed array of investigations in order to reach a definitive diagnosis. Finally, the report revealed the challenges in dealing with an active child athlete whose lifestyle would be limited due to this condition and may regard historical sport-related impacts as insignificant. Such a patient requires careful management to optimise their outcomes, minimise complications and allow a return to full activity.

Diagnosis of MO may be difficult and often requires radiological and, occasionally, histological confirmation.^[4]^ Several blood tests were initially performed on this patient, namely, full blood count, urea, electrolytes and creatinine, C-reactive protein, erythrocyte sedimentation rate, and creatine phosphokinase. The findings were unremarkable, apart from a moderate thrombocytosis. An MRI revealed a heterogeneously enhancing mass with thin peripheral calcification within the right gluteus maximus, in keeping with the findings of myositis ossificans described in the literature. Osteosarcoma is a possible differential and must be excluded before confirming the diagnosis of MO. Radiology is insufficient in differentiating between the two, necessitating a biopsy.^[2]^ The patient’s results yielded no histological evidence of malignancy and the findings were consistent with MO, confirming the diagnosis.

Physiotherapy and sporting activities were stopped to prevent further exacerbation of the lesion. Initial treatment of MO aims to reduce pain and inflammation. Indomethacin is recommended as prophylaxis and was prescribed in this case. It has been shown to reduce the extension of MO by inhibiting COX-1 and COX-2 enzymes, which play a role in regulating the differentiation of mesenchymal stem cells into osteoblasts.^[5]^ Surgical excision and radiation were not indicated in this case. Surgery is only considered necessary if there is no improvement observed after 6 – 12 months of alternative management. Radiation is generally avoided in children due to concerns about the potential carcinogenic effects it might have on the patient’s life in the future.^[2]^

Extracorporeal shock-wave therapy involves using single sonic pulses of short duration to induce a mechanical cavitation effect, not only fragmenting heterotopic calcification but also stimulating biological tissue repair.^[6]^ It has been shown to have significant analgesic and anti-inflammatory effects within a few weeks.^[6]^ The procedure is minimally invasive and has few side effects, such as bruising, short-term swelling and tenderness.^[6]^

This patient was successfully managed after two months of the conservative management, as evidenced by stabilisation followed by reduction in size of the lesion (see Figure 3). Her pain was dramatically reduced, enabling her to return to functioning normally.

## Conclusion

MO is rare and may resemble malignancy, necessitating a thorough workup. A high index of suspicion can avoid mismanagement and unnecessary treatment. This case of MO followed sport-related blunt force trauma and presented with the typical clinical, radiological, and histological findings of MO. The patient was managed successfully with conservative treatment, including NSAIDs and ESWT. This case highlighted the importance of a thorough history, accurate diagnosis, and the significant impact ESWT can have on the outcome of MO.
